# Social Disparities in Helmet Usage in Bicycle Accidents Involving Children

**DOI:** 10.7759/cureus.42017

**Published:** 2023-07-17

**Authors:** Imani H Sweatt, Candace Barr, Aaron Gelinne, Alice Woolard, Carolyn Quinsey

**Affiliations:** 1 General Surgery, University of North Carolina at Chapel Hill, Chapel Hill, USA; 2 General Surgery, Penn State University College of Medicine, Milton S. Hershey Medical Center, Hershey, USA; 3 Neurosurgery, University of North Carolina at Chapel Hill, Chapel Hill, USA; 4 Genetics Curriculum, University of North Carolina at Chapel Hill, Chapel Hill, USA; 5 Neurological Surgery, University of North Carolina at Chapel Hill, Chapel Hill, USA

**Keywords:** head injury, insurance, gender, race, helmet use, bicycle accidents, social determinants of health

## Abstract

Background

Bicycle helmet use has a known protective health benefit; yet, pediatric populations have suboptimal helmet rates, which increases the risk of severe injuries. It is imperative to have an updated assessment of behavioral social disparities and for providers to be aware of them to better counsel patients. The study objective was to identify social determinants correlated with helmet use in children involved in bicycle accidents. Based on previous literature, we hypothesized that higher socioeconomic status, female sex, and Caucasian race were associated with increased helmet use.

Methods

A retrospective case series of 140 pediatric cases of bicycle-related traumas assessing helmet status. Participants presented to the emergency room with injuries due to a bicycle-related trauma and were subsequently admitted to the University of North Carolina (UNC) Hospital System in Chapel Hill, North Carolina (NC), from June 2006 to May 2020. The Institutional Review Board (IRB) approved study comprised a retrospective chart review of 140 cases from the pediatric (<18 years of age) trauma database with coding indicating bicycle-related injury. Zip codes were used to approximate the median household income utilizing the Proximity One government database. The primary exposure was helmet status, which was determined from the electronic record chart review. The hypothesis was formulated before the start of the study. The main outcomes measured in the study included age, sex, race, helmet status, zip code, insurance status, injury types, and mortality.

Results

There were a total of 140 study participants, of which 35 were female and 105 were male. Males comprised 79.6% of the non-helmeted group, while females were in the minority in both helmet status groups, with 65.7% still being non-helmeted. Additionally, 51.9% of patients who were helmeted used private insurance, and 59.3% of those non-helmeted used public insurance. Of the 71 head injuries, 88.7% were non-helmeted. Principally, this study found that 80.7% of children involved in a bicycle-related accident were not helmeted.

Conclusions

Despite NC legislation mandating that children under 16 years of age wear helmets while operating bicycles, many children injured in bicycle-related trauma are not complying with this requirement. This study demonstrates that specific populations have decreased rates of helmet usage and emphasize the continued need to monitor helmet behaviors.

## Introduction

For many decades, bicycle safety has been both heavily researched and publicized. A recent retrospective analysis by McAdams et al. reported that over 2.2 million pediatric patients (aged five to 17 years) were treated in US emergency departments for bicycle-related injuries over 10 years between 2006 and 2015. Of those injuries, 15% were head and/or neck injuries, with children aged 10 to 14 years being the most affected [1]. Additionally, 11% of total injuries were traumatic brain injuries [1]. McAdams et al. demonstrated children who wore helmets were less likely to have head or neck injuries and less likely to be hospitalized [1]. Scott et al. analyzed information from the 2002-2012 National Trauma Data Bank and supported this conclusion after finding that 78% of their study participants who had head/neck injuries were not helmeted. There was also evidence of increased mortality risk, hospital stay length, and intensive care unit stay length among those who were not wearing helmets [2]. In efforts to minimize head, neck, and brain damage from potential cycling accidents, the use of bicycle helmets is highly recommended and, in many states, mandated by law for children under the age of 18 years [1].

Despite the evidence of the effectiveness of helmet use in injury prevention, a large proportion of cyclists do not wear helmets. Reportedly, half of all bicyclists wear a helmet for *some* trips and only 35% wear one for *all or most* trips [3]. Regarding children under 18, there is no clear consensus as to how often they are wearing helmets. One study determined approximately 69% of children under the age of 16 wore a helmet regularly, but this value was reported by parents [4]. Another study found only 15% of children under the age of 15 wore helmets *all or most of the time* in the United States [5]. Despite these discrepancies, the current body of relevant literature supports the finding that children use helmets at suboptimal rates.

Macpherson et al. conducted a retrospective study and subsequent stratified analysis to assess economic disparities in bicycle helmet usage in children. Their aim was specific in looking at the effect of helmet usage after the introduction of new legislation mandating protective wear for all cyclists in the East York health district of Canada [6]. While initial helmet use rose in all income levels, by six years post-legislation, usage by children in low- and middle-income areas had reduced to 33% and 50%, respectively [6]. Across every year analyzed, children from higher-income families were found to be 3.4 times more likely to ride with a helmet compared to children from lower-income backgrounds [6].

A study published by Kraemer in 2016 sought to examine if bicycle helmet laws were impactful in the reduction of existing racial disparities with helmet use. Using data collected from the 1991-2013 Youth Risk Behavior Survey, it was found that while helmet use increased across all racial groups, the greatest increases were among Caucasian students [7]. Kraemer also suggested that public policymakers take steps to address and help reinforce the need for helmet safety. In addition to determining if current legislation is reinforcing disparities, the study suggested assessing infrastructural issues that make cycling conditions more dangerous regardless of helmet usage.

A research project based in Winnipeg, Manitoba, aimed to increase helmet use among urban and rural communities [8]. Investigators found that 26.3% of females, regardless of age, used helmets consistently, while 18.9% of their male counterparts used helmets regularly [8]. They also identified adolescents and those from low-income areas as more likely to not wear bicycle helmets [8]. Prati et al. further examined the differential experience between male and female cyclists who had crashed. They found that the higher incidence of traumatic injuries could be attributed to a variety of factors, including a propensity for riskier behavior (e.g., lack of helmet use), cycling locations, and general road safety [9].

Though most of the present literature leads us to believe certain societal or demographic factors are correlated with increased preventative care, a recent development with seatbelt use should also be taken into consideration. Previous work has suggested that adults with less than a high school education are less likely to use seatbelts while in vehicles and that low-income families are more at risk for not using seatbelts for children [10]. However, the Safe Kids Worldwide Study found evidence that higher-income parents were least likely to properly restrain their children in vehicles when compared to both low- and middle-income parents [10]. It is important to note trends that could indicate a change in behavior toward protective wear. Whether the hypotheses are proven or disproven, it will be important to release the most up-to-date information to the public.

Among pediatric patients admitted for bicycle-accident-related injuries, we hypothesize the following: patients from higher socioeconomic backgrounds, female patients, and Caucasian patients are more likely to have worn a helmet. This study aims to identify current helmet rates in children who have been admitted to the hospital for bicycle-related trauma and assess demographic determinants of helmet usage within this same population. We will be evaluating helmet practices as a preventive health measure and identifying the most current at-risk groups.

## Materials and methods

We conducted a retrospective case series from the UNC Hospital system in Chapel Hill, North Carolina, from June 2006 to May 2020, from the pediatric (<18 years of age) trauma database with coding indicating bicycle-related injury. The primary International Classification of Diseases, 9th Revision (ICD 9) codes were 813.6, 814.6, 814.7, 816.6, 819.6, 826.1, 826.8, 884.9, and 888.1. We reviewed cases where a patient reported to UNC Hospitals following a bicycle-related accident, which could involve being struck by a vehicle while on a bicycle or a bicycle crash. Cases involving motorized bicycles were excluded from this review. We then determined whether the patient was wearing a bicycle helmet when the accident occurred. Cases with unknown helmet status were recorded as such. After Institutional Review Board (IRB) approval in June 2020, we collected information about patient age, sex, race, helmet status, zip code, injury types, surgeries, disposition, and mortality. Zip codes were used to determine median household income based on the Proximity One government database. The study was approved by the IRB on June 2020 and conducted by human subject guidelines.

Data were analyzed in R version 4.1.3, RStudio v 2021.09.0 (Posit, Boston, MA, USA). Logistic regression analysis was used to compare demographic characteristics and helmet status. Odds ratios (ORs) were calculated for sex, insurance type, and income status. An alpha value of 0.05 was used as the significance cutoff for *P*-values.

The study was granted formal approval by the IRB in June 2020 under the exempt review criteria. Only medical record data relevant to the study were obtained to not adversely affect the rights or welfare of the subjects. All data were securely coded and stored in an anonymous and de-identified process. 

## Results

Of the 171 cases reviewed from the pediatric trauma database, 140 cases met the criteria to be included in the study. Cases with unknown helmet status were excluded from the study. Overall, 35 participants were female and 105 were male (Table 1). It was found that Caucasian children made up the majority of cases with 82 participants (Table 1). Additionally, there were 33 African-American, 17 Hispanic, and eight *Other* identifying children (Table 1). Participants labeled as *Other* did not have a record of a specific race noted in the electronic chart. Of the 140 cases, 52 involved a motor vehicle and 88 was a bicycle crash (Table 1). The insurance status overall was as follows: 76 were publicly insured, 42 were privately insured, and 22 with no insurance at the time of the accident (Table 1). Examining all cases, the helmet status is as follows: 27 helmeted and 113 not helmeted.

**Table 1:** Study demographics, variables, and associated helmet status. Table demonstrating demographics of study participants and associated helmet status. Collision type, loss of consciousness, and recorded injury type with helmet status are also listed. The percentage of participants within helmeted and non-helmeted groups was also reported. ^a^Wilcoxon rank-sum test; Fisher’s exact test; Pearson’s chi-square test. ^b^Median (IQR) ^c^*n* (%). IQR, interquartile range

Characteristics	Helmet status		*P*-value^a^
	No (N = 113)	Yes (N = 27)	
Age at admission^b^	10 (8-13)	10 (8-14)	0.6
Race^c^			0.017
African American	29 (26%)	4 (15%)	
American Indian/Alaskan Native	7 (6.2%)	0 (0%)	
Caucasian	59 (52%)	23 (85%)	
Hispanic	17 (15%)	0 (0%)	
Other	1 (0.9%)	0 (0%)	
Sex^c^			0.009
Female	23 (20%)	12 (44%)	
Male	90 (80%)	15 (56%)	
Insurance status^c^			0.020
None	18 (16%)	4 (15%)	
Private	28 (25%)	14 (52%)	
Public	67 (59%)	9 (33%)	
Collision type^c^			0.074
Bicycle only	67 (59%)	21 (78%)	
Motor vehicle collision	46 (41%)	6 (22%)	
Loss of consciousness^c^			0.6
No	65 (58%)	17 (63%)	
Unknown	5 (4.4%)	2 (7.4%)	
Yes	43 (38%)	8 (30%)	
Head injury^c^			0.015
No	50 (44%)	19 (70%)	
Yes	63 (56%)	8 (30%)	
Head/neck injury			0.14
No	62 (55%)	19 (70%)	
Yes	51 (45%)	8 (30%)	
Spine injury^c^			0.4
No	107 (95%)	24 (89%)	
Yes	6 (5.3%)	3 (11%)	

**Figure 1:**
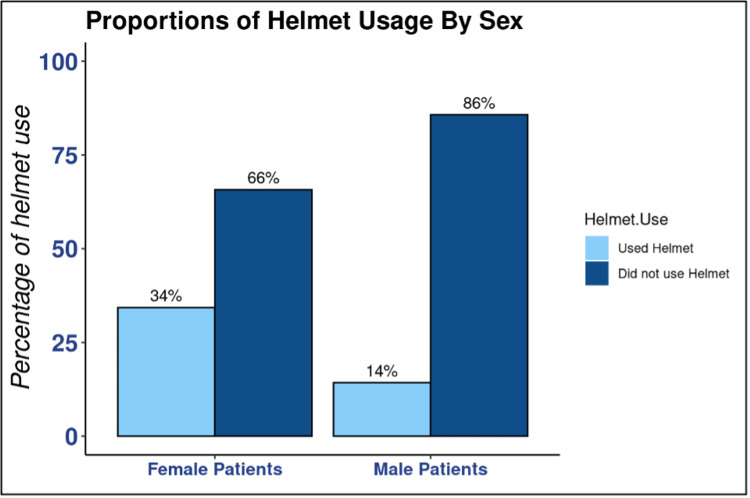
Proportions of helmet usage by patient’s sex. Bar graph demonstrating the percentage of helmet usage by patient’s sex as reported in the electronic medical record. Male patients had a higher percentage of participants with a “Not Helmeted” status when compared to female patients. However, both groups had higher percentages of a “Not Helmeted” status.

**Figure 2:**
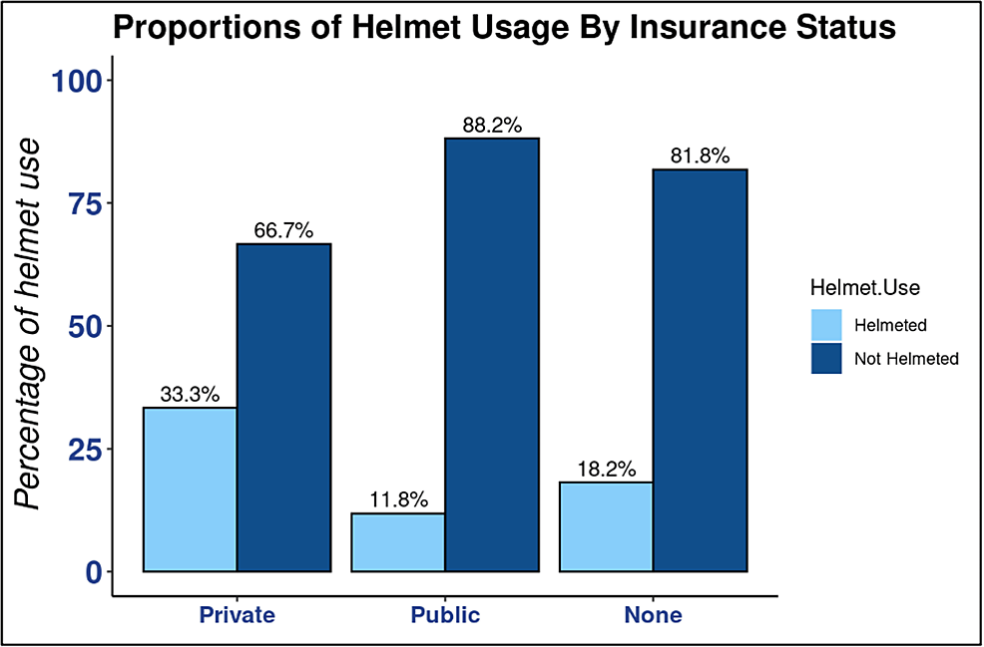
Proportions of helmet usage by insurance status. Bar graph demonstrating the percentage of helmet use by insurance status reported in the electronic medical record at the time of the injury. Patients with public insurance had the highest percentage of “Not Helmeted” status compared to those with private or no insurance. However, all groups had a percentage greater than 65% with a “Not Helmeted” status.

**Figure 3:**
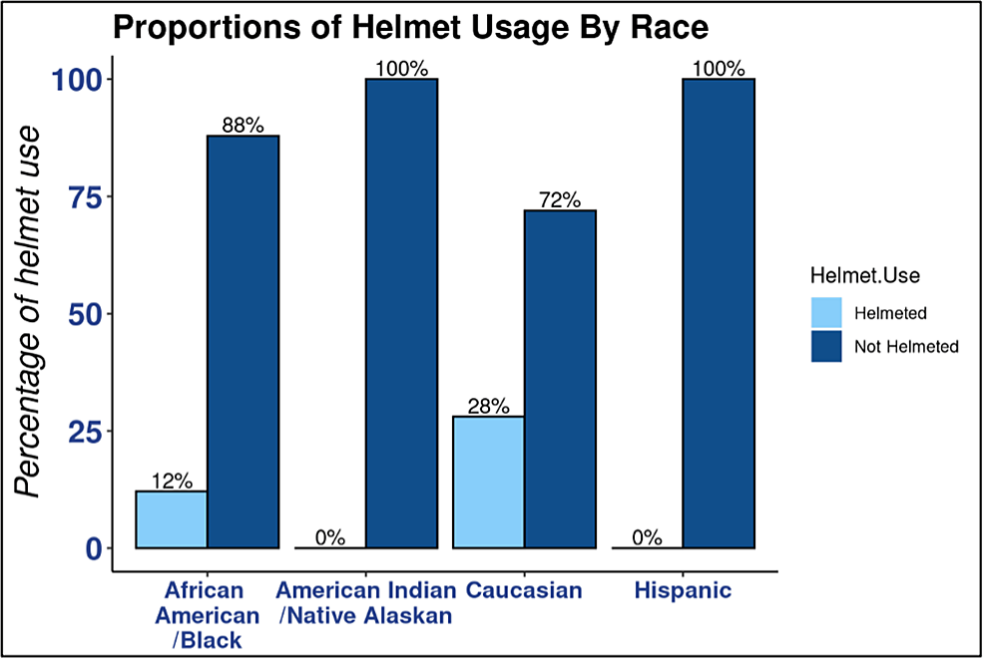
Proportions of helmet usage by race. Bar graph demonstrating the percentage of helmet usage by race reported in the electronic medical record. Every group was found to have a higher percentage of patients with a “Not Helmeted” status. Notably, patients from classically underrepresented minority groups had higher percentages of those with a “Not Helmeted” status when compared to Caucasian patients.

We found that 80.7% of children involved in a bicycle-related accident in this study were not helmeted. Males made up 79.6% of the non-helmeted group (*P *= 0.009) (Table 1). While females were in the minority in both helmet status groups, 65.7% were still non-helmeted (*P *= 0.012) (Figure 1). When comparing males to females, male participants were less likely to be helmeted (OR 0.32, 95% confidence interval [CI] 0.13-0.78, and *P *= 0.012) (Figure 1). It is important to note that males outnumbered females (105:35). Regarding insurance status, 51.9% of patients who were helmeted used private insurance (*P *= 0.018), and 59.3% of those non-helmeted used public insurance (*P *= 0.018) (Figure 2). Comparing the two insurance statuses, publicly insured patients were less likely to be helmeted (OR 0.27, 95% CI 0.10-0.68, and *P *= 0.006) (Figure 2). Of the 71 documented head injuries with known helmet status, 88.7% were non-helmeted (*P *= 0.015). Of the non-helmeted patients, 55.8% experienced a head injury (*P *= 0.015). Of the total patients wearing a helmet, 70.4% did not have a head injury (*P *= 0.015). When assessing race, we found that 85.2% of the helmeted group was Caucasian (*P *= 0.011) (Figure 3). However, 52.2% of the non-helmeted group was also Caucasian and 25.7% was African-American (*P *= 0.011) (Figure 3).

We also assessed a subset of 52 cases of children who were involved in vehicular-related accidents with known helmet status. In this subset, 46 (88.5%) children were not helmeted. 

## Discussion

Wearing a helmet has proven to be a necessary safety measure to protect against potential head trauma while operating a bicycle. Research demonstrating this correlation along with reported low rates of helmet usage after the enactment of safety laws mandating children wear bicycle helmets [11]. Our study sought to assess current helmet usage among hospital-admitted pediatric patients and identify any potential social determinants affecting such usage. The results were alarming, as our data indicate that four out of every five children were not wearing helmets during the injury leading to their admission. As hypothesized, various social factors correlate with higher percentages of non-helmet status.

Supporting our initial hypothesis, we found that male children were less likely to wear a helmet compared to their female counterparts. These patients were also more likely to be involved in bicycle-related accidents in general. This could be explained by the notion that boys may be more prone to engage in riskier behavior than girls [9]. While not as high a rate as males, female patients were still more likely than not to forego a helmet in their injury-causing accident. However, it is important to note that there were three times the number of male participants compared to females, and this could indicate sampling bias. 

Our data also supported our hypothesis that Caucasian children were wearing helmets at higher rates compared to other children in the other racial groups. Yet, Caucasians still made up more than half of those found to be non-helmeted, which was more than any other racial group. This result is interesting as it supports the notion that safety guidelines may be reaching this population more effectively, but helmet usage is not necessarily the standard. Further analysis needs to be done on economic status before definitively assessing that aspect of our hypothesis.

This study is limited by both its retrospective nature and some of our data collection methods. Due to the variability in provider notetaking, we are unable to outline one specific technique for abstracting information. For example, some cases had all of the needed information in a history and physical note, while others had information in a triage note. Furthermore, there were 31 individuals with an unknown helmet status whose inclusion in the full analysis of the data may have provided further insights into at-risk groups. It should also be noted that the data were collected from charts over 14 years, so changes in notetaking may have also affected data extraction.

It is also important to recognize the possibility of confounders when assessing social disparities. There may be variables that affected children in specific subgroups and their usage of helmets. Regarding race, there were individuals whose race was not identified or self-identified as other. It is unclear if the provider or patient might make this distinction or the reason why. Though only eight individuals made up that group, there is the possibility that those cases should have been relegated to a specific racial group. Additionally, while the use of zip codes has been used historically to assess household income, it is still only an estimate and not necessarily the most accurate representation.

The scope of this study was limited to patients who presented to the emergency room and then admitted to the hospital. Therefore, our analysis does not allow for a true assessment of helmeting rates or helmet safety in all children cyclists. There is a possibility that more children would have been included in the study, but their injuries were not severe enough for a hospital visit. However, noting the increased severity of the included cases suggests the safety benefits of helmet use.

Our study, by previous literature, demonstrates that there is a significant protective benefit with helmet compliance and thus a critical need to assess helmet usage [1,12]. As time progresses, future studies will be necessary to assess the evolution of helmeting behaviors, particularly after the implementation of legislation and community efforts aimed at increasing helmet usage. There are at-risk groups with lower helmet rates, which also indicates the importance of further understanding why such disparities are persisting and to monitor their development.

## Conclusions

The results of this study provide updated information and context about the current helmet practices among pediatric patients receiving emergency care for bicycle-related trauma. While there is potential for sampling bias based on gender, our study found that females, Caucasians, and privately insured patients were more likely to be helmeted. Our analysis supports and expands upon the current body of literature assessing pediatric helmet usage, giving policymakers and providers insights into the demographics of these patients. Critically, this study and future investigations are needed to determine the optimal approaches for serving the needs of at-risk populations. 
